# A single-centre study on predictors and determinants of pubertal delay and growth impairment in Epidermolysis Bullosa

**DOI:** 10.1371/journal.pone.0274072

**Published:** 2022-09-06

**Authors:** Giulia Rodari, Sophie Guez, Simona Salera, Fabio Massimo Ulivieri, Gianluca Tadini, Michela Brena, Eriselda Profka, Federico Giacchetti, Maura Arosio, Claudia Giavoli

**Affiliations:** 1 Fondazione IRCCS Ca’ Granda Ospedale Maggiore Policlinico, Endocrinology Unit, Milan, Italy; 2 University of Milan, Department of Clinical Sciences and Community Health, Milan, Italy; 3 Fondazione IRCCS Ca’ Granda Ospedale Maggiore Policlinico, Pediatric Highly Intensive Care Unit, Milano, Italy; 4 Centre for Diagnosis and Treatment of Osteoporosis, Casa di Cura La Madonnina, Milan, Italy; 5 Fondazione IRCCS Ca’ Granda Ospedale Maggiore Policlinico, Pediatric Dermatology, Pediatric Highly Intensive Care Unit, Milano, Italy; University of Freiburg, UNITED STATES

## Abstract

**Background:**

Delayed puberty is a possible complication of Epidermolysis Bullosa (EB), though the actual incidence is still unknown. In chronic illnesses delayed puberty should be correctly managed since, if untreated, can have detrimental effects on adult height attainment, peak bone mass achievement and psychological health.

**Aims and methods:**

This is a single-centre study on pubertal development, growth and bone status in EB. Auxological, densitometric (areal Bone Mineral Density-aBMD Z-score, Bone Mineral Apparent Density-BMAD Z-score, Trabecular Bone Score-TBS and Bone Strain Index-BSI at Lumbar spine) and body composition data (Total Body DXA scans) were collected. Disease severity was defined according to Birmingham Epidermolysis Bullosa Severity (BEBS) score.

**Results:**

Twenty-one patients (12 Recessive Dystrophic EB-RDEB, 3 Dominant Dystrophic EB, 3 Junctional EB-JEB, 2 EB Simplex and one Kindler EB) aged 13 years (females) or 14 years (males) and above were enrolled (age 16.2±2.5 years, M/F 11/10). Short stature was highly prevalent (57%, mean height -2.12±2.05 SDS) with 55% patients with height <-2SD their mid-parental height. 7/21 patients (33%, 6 RDEB and 1 JEB) had delayed puberty with a median BEBS of 50 (range 29 to 63), a height SDS of -2.59 SDS (range -5.95 to -2.22) and a median lumbar BMAD Z-score of -4.0 SDS (range -5.42 to -0.63 SDS). Pubertal status was negatively associated with BEBS, skin involvement, inflammatory state and positively with height SDS and BMI SDS.

**Conclusions:**

Pubertal delay is highly prevalent in EB, especially in patients with RDEB and JEB, high severity score and inflammatory state. Moreover, pubertal delay worsens growth impairment and bone health. A study on pubertal induction is ongoing to enlighten possible beneficial effects on adult height attainment and peak bone mass accrual.

## Introduction

Epidermolysis bullosa (EB) is a group of extremely rare mechanobullous genodermatoses characterised by varying degrees of skin and mucous membrane fragility, which readily blisters in response to minor mechanical trauma [1]. The underlying pathogenic mutations of hereditary EB involve genes of structural proteins within the epidermis, skin basement membrane zone (dermo-epidermal junction), or uppermost dermis [1]. Although EB is often viewed as a skin disease, the most severe forms, such as Recessive Dystrophic EB (RDEB) or Junctional EB (JEB), are actually multisystemic disorders with extracutaneous complications, such as oesophageal strictures, cardiomyopathy, anaemia, growth failure and reduced bone mass [2–7].

Delayed puberty, usually defined as the absence of any pubertal development at an age of 2 standard deviations (SD) more than the mean (approximately 14 years for boys and 13 years for girls), represents a common complication of EB, though the actual incidence is still unknown [8]. The timing of pubertal onset is still a mysterious and complex phenomenon, indeed the actual mechanisms underlying the great interindividual variation are yet to be defined. Moreover, pubertal timing shows a familial pattern and seems to be largely defined genetically, whereas environmental factors may influence this genetic regulation and can possibly explain as much as 25% of the interindividual variation. However, in recent years a secular trend towards earlier puberty has been observed, probably due to nutritional status and obesity [9].

In chronic illnesses, delayed puberty is often considered a consequence of the underlying disease itself, however it should be correctly managed since today a great number of children affected by these conditions can survive far beyond the age of physiological puberty [10]. Disease severity, age of onset and duration are usually considered the most important predictors of pubertal delay, as well as malnutrition and inflammatory state, which are considered other major possible causative factors [8]. However, no data on EB children are currently available to support the association between disease severity, inflammatory state and pubertal delay.

The aim of the present single-centre study is to investigate pubertal development, growth and bone status in EB in order to better define predictors and determinants of pubertal delay and short stature.

## Methods

This is an observational cross-sectional study on 44 children (M = 25, 57%) regularly followed at the Center for Epidermolysis Bullosa in Northern Italy at Fondazione IRCCS Ca’ Granda Ospedale Maggiore Policlinico, Milan. Among them, 21 patients referring to the Endocrine clinic between January 2020 and December 2020, with at least 13 years (females) and 14 years (males), were included. Clinical phenotype, skin biopsy and genetic mutation analysis (available for all study patients) supported the diagnosis in accordance with the latest Consensus report [1]. Parental written informed consent was obtained from parents; moreover, children ≥12 years were required to give their written assent.

Ethical approval was waived by the local Ethics Committee of Fondazione IRCCS Ca’ Granda Ospedale Maggiore Policlinico of Milan. All the procedures being performed were part of the routine care.

History and physical examination were collected for each patient. Height (HT), mid-parental height (MPH), weight, body mass index (BMI) and pubertal stage were recorded and SDS values for HT and MPH were calculated according to the Italian reference charts for Italian patients (Cacciari Growth Charts) [11] and WHO growth charts for the only two non-Italian patients (from Albania and Croatia) according to “Growth calculator 4” by Italian Society of Paediatric Endocrinology and Diabetology (ISPED-SIEDP) available online [12]. Body mass index SDS was evaluated according WHO specific charts [12, 13]. In RDEB and JEB patients, for whom disease-specific growth charts are available, height and BMI were also considered in comparison with patients with the same age, gender and form of EB [5]. Short stature was defined as HT < - 2 SDS. Pubertal development was assessed by Paediatric Endocrinologists and compared with Tanner stages.

Delayed puberty was considered as the absence of any pubertal development at an age of 2 standard deviations (SD) more than the mean, namely testicular volume <4 mL at 14 years for boys and lack of breast budding at 13 years for girls [14].

Mid-parental height (MPH) was calculated according to the following equations [15]:

motherheight−fatherheight+132inboysmotherheight−fatherheight−132ingirls

Mid-parental height distance was defined as MPH-HT SDS < - 2 SDS.

Bone age (BA), as calculated according to the standards of Tanner-Whitehouse [16], was obtained in 9/21 children. This data was not available in RDEB patients with pseudo-syndactyly and severe hand scars or older patients with completed epiphyseal fusion (12/21).

EB severity was evaluated following the Birmingham epidermolysis bullosa severity (BEBS) score, one of the available scoring systems which includes not only the area of skin damage but also nail and mucosal involvement, scarring of hands, skin cancer, chronic wounds present for at least 6 months, alopecia, and nutritional impairment. The term “area of skin damage” included blisters, erosions, scabs, healing skin, erythema, and atrophic scarring and excluded skin changes not resulting directly from damage, such as mottled pigmentation in Epidermolysis Bullosa Simplex (EBS) and poikiloderma in Kindler EB (KEB) [17]. This score, differently from more recent ones, not distinguishing between activity and damage, better reflects the disease involvement in a particular period of time. As for laboratory parameters, C-reactive protein (CRP), insulin-like growth factor-1 (IGF-I), luteinizing hormone (LH), follicle stimulating hormone (FSH), 17 β-oestradiol (in females) and testosterone (in males) levels were collected for each patient in the morning after overnight-fast. For the two girls with regular menses, random LH, FSH and 17 β-oestradiol samples were obtained under the same conditions.

Dual energy X-ray absorptiometry (DXA) scans of the lumbar spine (LS) and total body (TB) were available for all study patients (Hologic Discovery bone densitometers-software version 3.3 APEX, Discovery A, Hologic Inc., Marlborough, MA, USA). Lumbar spine bone mineral apparent density (BMAD, g/cm^3^) was also determined, thus obtaining a potential correction method in an attempt to approximate the true volumetric density in children with short stature, based on the assumption that the vertebral body is a cube: a fundamental correction in chronic illnesses in which growth failure is highly prevalent [18]. Age- and gender-adjusted standard deviation score (SDS) for BMAD L1–L4 were obtained following the equation and normative data provided by Crabtree et al. [18, 19].

For an appropriate assessment of bone fragility and fracture prediction, it is important to take into account, not only quantitative information on bone mineral density, but also qualitative data such as bone texture, geometry and bone strength. That is even more significant in chronic illnesses, where bone quality impairment can be quite relevant.

In order to add indirect information of bone microarchitecture with the only use of non-invasive methods, trabecular bone score (TBS) was also obtained and recorded [20].

Bone Strain Index (BSI) was calculated from LS DXA scans to provide evidence of the average equivalent strain inside the bone, with the assumption that a higher strain level (high BSI) stands for a more significant risk condition [21].

### Statistical analysis

Statistical analysis was performed using SPSS version 26 statistical package (SPSS IBM, New York, USA).

Descriptive analysis was used to characterize the study population (mean and standard deviation for normally distributed continuous variables, median and range for others). To compare two normally distributed continuous variables, Student’s t test was used; otherwise, a Mann–Whitney test was employed. Linear regression analysis was used to measure the relationship between Tanner stages and some auxological, clinical and laboratory variables (BEBS score, skin damage, HT SDS, MPH difference, BMI SDS, CRP).

Moreover, linear regression analysis was performed to investigate the predictors of low BMAD, BMAD Z-score, TBS and BSI between various clinical, auxological and laboratory variables (BEBS score, skin damage, HT SDS, MPH distance, BMI SDS, pubertal stages, CRP, IGF-I SDS, LH, FSH, 17 β-estradiol and total testosterone).

Statistical significance was defined as a two-sided *P*<0.05.

## Results

We analysed data of 21 patients with at least 13 years and 14 years (for females and males, respectively). Among them, 12 were affected with RDEB (9/12 severe and 3/12 intermediate), 3 with Dominant Dystrophic Epidermolysis Bullosa (DDEB), 3 with JEB (2/3 intermediate and 1/3 with pyloric atresia), 2 with EBS (1/2 severe and 1/2 localised) and one with KEB.

The main clinical characteristics of the study population (M11, 52%, aged 16.2±2.5 years, range 13.0 to 20.7) are reported in Table 1. Laboratory and densitometric data are reported in Tables 2 and 3, respectively.

**Table 1:** Auxological and clinical variables.

	CA^1^ (years)	Height (SDS)	MPH^2^ (SDS)	Height-MPH^2^ (SDS)	BMI^3^ (SDS)	BEBS^4^	BEBS^4^ skin
**RDEB [mean(SD)]**	16.8	-2.88	0.06	-2.95	-3.35	36.4	15.7
	(2.6)	(2.24)	(0.94)	(2.03)	(1.92)	(23.1)	(10.8)
Other* **[mean(SD)—median (range IQ)]**	14.9	-1.10	-0.08	-1.15	-1.69	7.5	5.4
	(13.9–16.3)	(1.25)	(1.00)	(1.28)	(1.75)	(3.5–17.6)	(4.3)
**All patients [mean(SD)]**	16.2	-2.12	0.01	-2.23	-2.64	25.6	11.1
	(2.5)	(2.05)	(0.94)	(1.95)	(1.99)	(22.4)	(9.9)
**N**	**21**	**21**	**20**	**20**	**21**	**21**	**21**

^1^Chronological age;

^2^Mid-parental height;

^3^Body Mass Index;

^4^Birmingham Epidermolysis Bullosa Severity

*Dominant Dystrophic Epidermolysis Bullosa, Junctional Epidermolysis Bullosa, Epidermolysis Bullosa Simplex and Kindler EB.

**Table 2:** Laboratory data.

	LH^1^ (mIU/L)	FSH^2^ (mIU/L)	17β-oestradiol (ng/L)	total testosterone (ng/mL)	CRP^3^ (mg/dL)	IGF-I^4^ (SDS)
**RDEB [mean(SD) -median (range IQ)]**	1.3 (0.1–5.3)	3.0 (2.1)	12.8 (5–167)	0.1 (0.04–2.11)	6.7 (3.6)	-2.63 (-3.05–-1.58)
Other* **[mean(SD)—median (range IQ)]**	2.9 (2.0)	3.4 (1.4)	19 (5–134)	3.8 (1.3–6.2)	0.2 (0.1–1.0)	-1.30 (0.71)
**All patients [mean(SD)–median (range IQ)]**	2.4 (0.1–4.6)	3.2 (1.8)	59.1 (75.2)	2.5 (2.5)	3.7 (0.2–9.0)	-1.81 (1.22)
**N**	**20**	**21**	**10**	**11**	**14**	**21**

^1^Luteinizing hormone,

^2^follicle stimulating hormone,

^3^C-reactive protein,

^4^insulin-like growth factor-I

*Dominant Dystrophic Epidermolysis Bullosa, Junctional Epidermolysis Bullosa, Epidermolysis Bullosa Simplex and Kindler EB.

**Table 3:** Densitometric data.

	Lumbar Spine aBMD^1^ (g/cm^2^)	Lumbar Spine Z-score (SDS)	Lumbar Spine Z-score BA^2^	BMAD^3^ (g/cm)	BMAD^3^ Z-score	TBS^4^	BSI^5^	TBLH^6^ Z-score (SDS)
**RDEB [mean(SD)]**	43.0 (6.0)	-3.66 (2.6)	-2.59 (2.14)	0.186 (0.061)	-2.70 (2.43)	1.165 (0.154)	2.21 (0.77)	-2.94 (2.14)
Other* **[mean(SD)]**	51.8 (9.5)	-1.73 (1.73)	-0.90 (1.51)	0.200 (0.031)	-1.10 (1.39)	1.254 (0.118)	1.87 (0.27)	-1.59 (1.48)
**All patients [mean(SD)]**	46.8 (8.7)	-2.83 (2.4)	-1.99 (2.05)	0.192 (0.05)	-1.95 (2.09)	1.203 (0.144)	2.07 (0.63)	-2.37 (1.97)
**N**	**21**	**21**	**14**	**21**	**16**	**21**	**21**	**19**

^1^Areal Bone Mineral Density,

^2^Bone Age,

^3^Bone Mineral Apparent Density,

^4^Trabecular Bone Score,

^5^Bone Strain Index,

^6^Total Body Less Head

*Dominant Dystrophic Epidermolysis Bullosa, Junctional Epidermolysis Bullosa, Epidermolysis Bullosa Simplex and Kindler EB

As long as pharmacological anamnesis was concerned, only two RDEB patients were taking oral budesonide syrup obtained from nebulizer solution at 0.25 mg alternate days in the presence of oesophageal strictures. There were no other chronic medication to declare.

### Growth and nutrition

Short stature (HT SDS<-2) was found in 12/21 children (57%), with 11/20 (55%) patients showing a significant distance to their genetic determined growth potential (MPH-HT SDS<-2 SDS) and more than a half of patients showed low BMI (<-2.0 SDS in 13/21 children).

As far as nutrition is concerned, one RDEB patient was fed through a gastrostomy tube and three RDEB patients received fortified drinks. Though widely prescribed, only eight patients were taking vitamin D. Moreover, four patients were on iron, two on folic acid and one on zinc supplements.

Reflecting both the nutritional and pubertal status, IGF-I levels were often under the age- and sex-adjusted reference range (43%, 9/21 patients) as shown in Table 3.

### Puberty

From the clinical point of view, 6/21 patients (29%) were prepubertal (Tanner stage I with testicular volume TV 2–3 mL in males); the remaining had started pubertal development (Tanner stages II to V, for males TV 13.7±7.6 mL, from 4 to 25 mL). Among them, 7/21 patients (33%) had completed puberty (Tanner stage V).

Pubertal delay was found in 7/21 patients (33%, 6 RDEB and 1 JEB, 4F/3M): 4/10 (40%) girls and 3/11 (27%) boys, with no gender differences (*P* = 0.66). All of them were Tanner stage I at the time of assessment, with the only exception of one male patient who was 17 years old and Tanner stage III (TV 6 mL) but, as inferred from medical records, entered into puberty at 15 years of age (TV 4 mL). In the whole group with pubertal delay, median BEBS was 50, median HT SDS -2.59 SDS and median BMAD Z-score of -4.0.

**Fig 1:**
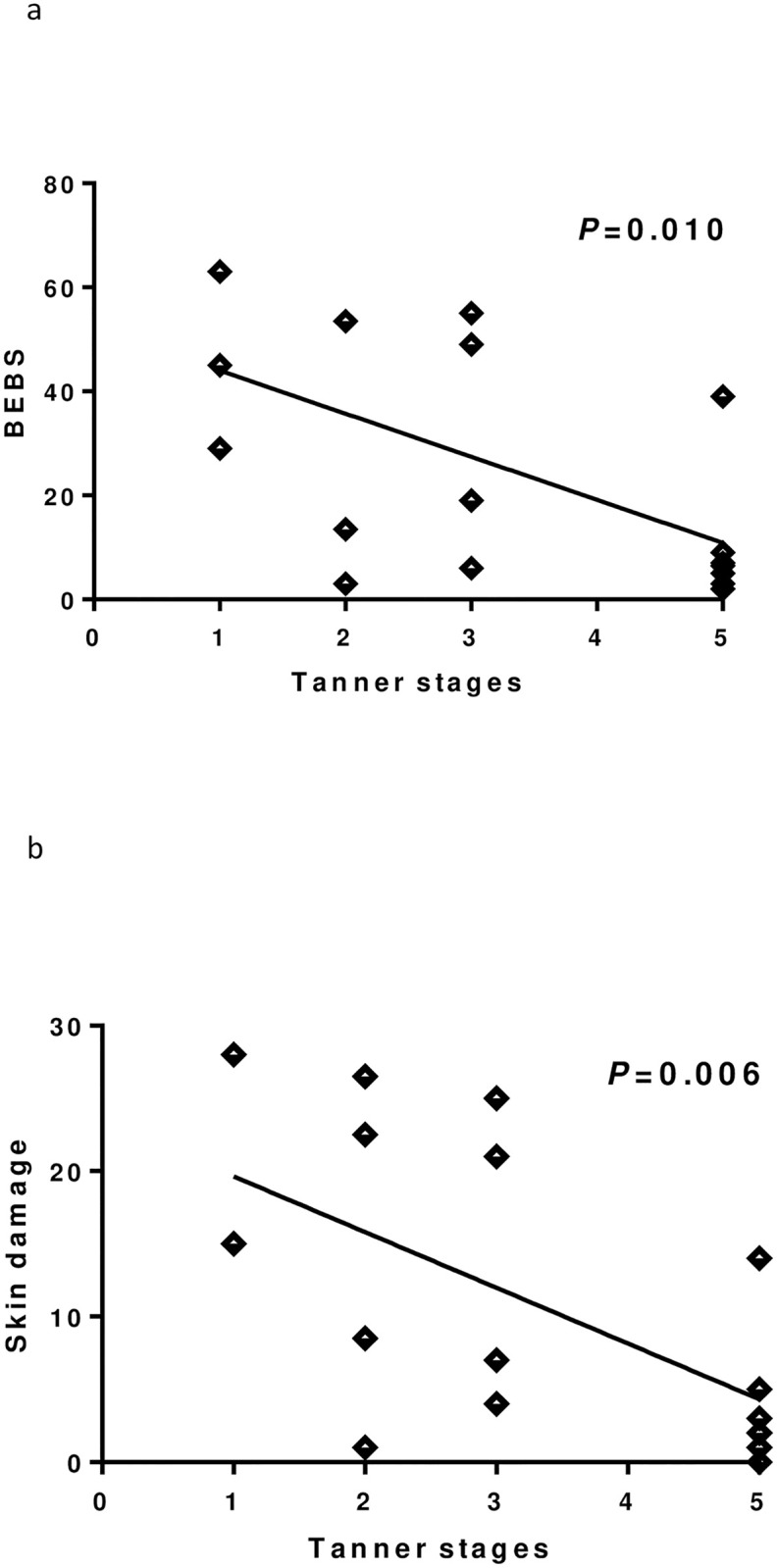
Linear regression between Tanner stages and disease severity. (a) Birmingham Epidermolysis Bullosa Severity (BEBS) score. (b) skin involvement.

Considering the different forms of EB, 6/12 patients with RDEB (50%) and 1/3 JEB (33%) showed delayed puberty (Fig 1).

### Bone status

As far as bone status is concerned, lumbar spine aBMD was reduced in 67% children (14/21 patients), remaining low in 44% even after correction for short stature.

Bone age was available for 9/21 patients (43%, 14.5±3.6 years) with a mean significantly different from chronological age (*P* = 0.022). X-ray of the hand and wrist was not performed in 12/21 children with pseudosyndactyly and severe hand scars or older patients with complete pubertal development.

**Fig 2:**
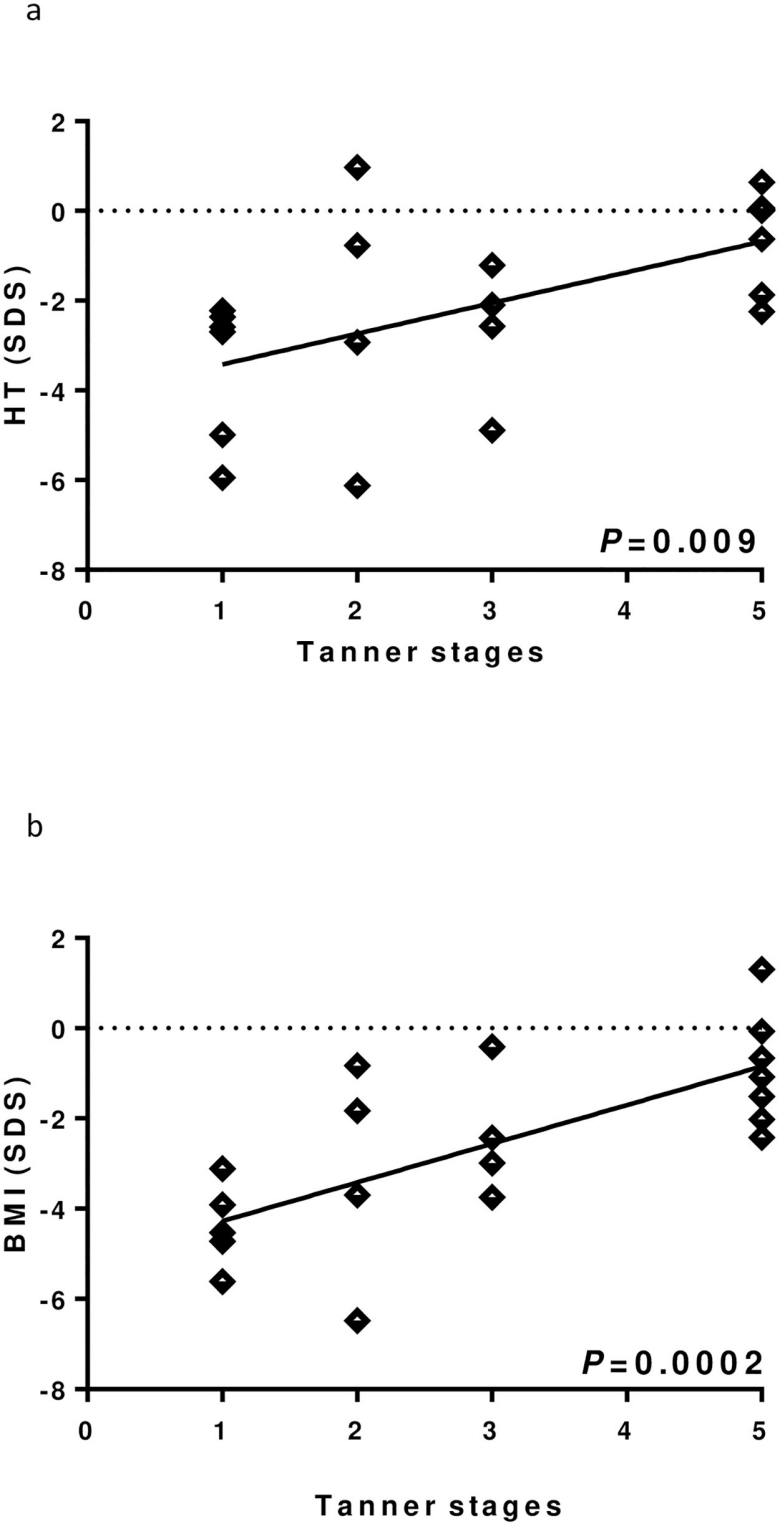
Linear regression between Tanner stages and auxological variables. (a) Height (HT) SDS. (b) Body Mass Index (BMI) SDS.

**Fig 3:**
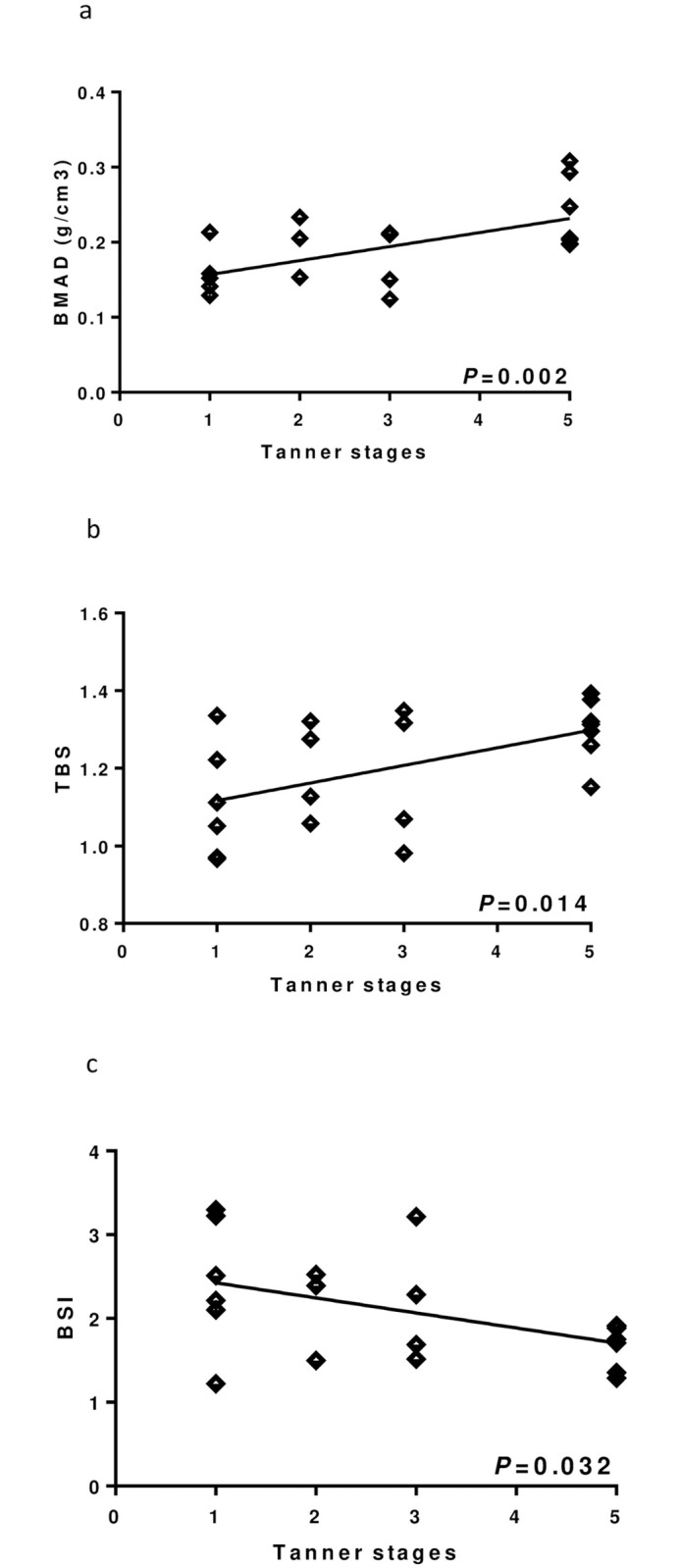
Linear regression between Tanner stages and bone mass. (a) Bone Mineral Apparent Density (BMAD). (b) Trabecular Bone Score (TBS). (c) Bone Strain Index (BSI).

At linear regression, pubertal stage was negatively associated with BEBS score (Fig 2a), skin damage (Fig 2b) and CRP while positively with HT SDS (Fig 3a) and BMI SDS (Fig 3b).

**Fig 4:**
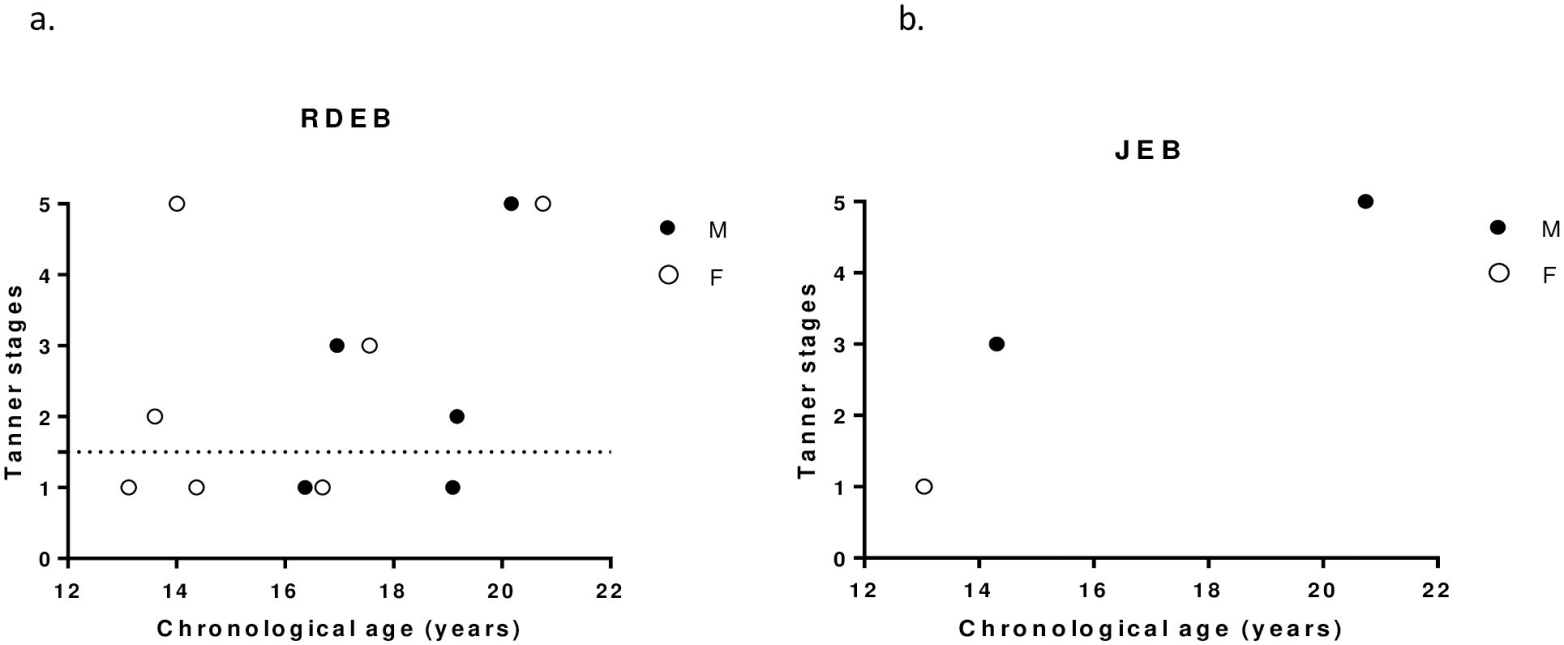
Tanner stages and chronological age (years) for patients with Recessive Dystrophic EB (a) and Junctional EB (b).

Furthermore, IGF-I levels were negatively correlated with BEBS score, skin damage and CRP but positively with MPH distance, HT SDS and BMI SDS. Moreover, as expected, BMAD, BMAD Z-score and TBS were negatively influenced by BEBS score and skin involvement while positively by HT SDS, MPH distance, BMI SDS, Tanner stages (Fig 4a and 4b), total testosterone in boys and IGF-I SDS. In girls, BMAD and BMAD Z-score were positively associated with 17 β-oestradiol whereas BMAD, BMAD Z-score and TBS were negatively associated with CRP levels. As far as BSI was concerned, high BSI was influenced by higher BEBS score, skin involvement, lower BMI SDS, HT in respect to MPH, Tanner stages (Fig 4c), total testosterone in boys and IGF-I SDS.

One patient with delayed puberty in RDEB showed two vertebral fractures at thoracic and lumbar spine X-ray performed for back pain at the age of 14.4 years (BMAD 0.152 g/cm^3^, -3.67 SDS, TBS 0.970, BSI 2.513).

## Discussion

The present study adds evidence that pubertal delay is highly prevalent in EB, especially when considering the most severe forms (i.e. RDEB), involving one third to a half of patients.

The definition of physiological puberty is based on a statistical criterion, namely if the onset of pubertal signs occurs within the range of ± 2.5 SDS for sex- and age-adjusted reference values. Historically, these values came from the pivotal epidemiological studies by Tanner and Marshall [22]. Even though a trend towards earlier puberty has been observed over the last decades, thus suggesting the need of a redefinition of the normal limits, up to now a consensus on this topic is still lacking [23].

Constitutional pubertal delay, with a reported prevalence of 2–2.5% [14, 24], stands for approximately 65% of all boys with delayed puberty. In the present cohort, no gender difference was found, suggesting that other underlying mechanisms must be taken into account when considering pubertal delay in the context of EB.

Delayed puberty may be secondary to poor nutrition, increased energy expenditure, systemic therapy for chronic conditions (especially long-term glucocorticoids) and proinflammatory state [25–27].

Malnutrition is highly prevalent in EB, with 56% of patients below the 3^rd^ centile for weight, with a higher incidence in JEB and RDEB [28], thus the importance of establishing a nutrition-based approach to the disease [29]. Malnutrition can indeed interfere with the entire hormonal system and, accordingly, the hypothalamo-pituitary-gonadal axis. Low body weight is associated with a delay in hypothalamic-pituitary-gonadal (HPG) axis maturation and GnRH pulse generator activation, postponing the onset of puberty and consequently impairing the “growth spurt” [8]. Even though the endocrine system has been poorly investigated in patients with EB, low body weight is likely to play one of the principal roles in delayed growth and puberty in these children.

An imbalance of pro-inflammatory cytokines, commonly found in inflammatory diseases (i.e. interleukin-1B –IL-1, tumour necrosis factor- TNF- and interleukin-6- IL-6), can alter HPG axis function. In particular, pro-inflammatory cytokines can directly inhibit hypothalamic secretion of GnRH or interfere with steroidogenesis in the ovary and the testis [30]. Pro-inflammatory cytokines (IL-1, TNF and IL-6) in EB patients have been found to be higher than healthy subjects, especially in patients with DEB [31, 32]. In the present cohort of EB children, the association between pubertal delay and low BMI, higher PCR values and increased disease severity (higher BEBS score and skin involvement) strengthen this concept. In fact, up to a half of our patients with RDEB, one of the most severe EB forms, showed delayed puberty.

In some chronic conditions, notably inflammatory bowel diseases, delayed puberty and growth, are thought to be related to malnutrition and the effects of cytokines on the GH/IGF-I axis [26]. It is well known that IL-6 tends to induce a relative hepatic GH resistance, by inducing SOCS-3 [33]. Moreover, it can increase the proteolysis of IGFBP-3 with impaired formation of the IGF-I/IGFBP-3/acid labile subunit complex, resulting in a shorter half-life and enhanced clearance of IGF-I. In addition, the cytokine TNF-alpha directly reduce hepatocyte expression of the GH receptors, thus lowering IGF-I levels [34]. Although no data are available on the GH/IGF-I axis in EB patients, we previously described profoundly reduced IGF-I (SDS) levels in EB children, with IGF-I lower than -2 SDS in 40% cases [5]. These results were confirmed even in this cohort by the negative correlation with CRP, BESB and skin involvement, supporting the possible interference of the inflammatory state and disease activity on GH/IGF-I axis.

Pubertal delay has been described in other chronic conditions, such as Duchenne Muscular Dystrophy, where the chronic use of glucocorticoids, inhibiting the HPG axis, may result in hypogonadotropic hypogonadism and, subsequently, testosterone deficiency [35–37]. Among our patients, the delay is unlikely related to the chronic use of steroids, given only in two patients with oesophageal strictures at dosages under the daily physiological cortisol secretion, for alternated days and as oral syrup from nebulizer solution.

Pubertal delay can negatively affect peak bone mass achievement [38] due to the permissive effects of sex hormones on mineralization. This is an aspect to take into consideration in order to prevent future risk of fracture in patients with a chronic disease associated with reduced BMD even during pre-pubertal period [5]. Though preliminary data and on a limited number of patients, our TBS and BSI results bring to light the qualitative bone involvement in EB. According to our data, the qualitative bone impairment strictly depends on disease severity, skin damage and nutritional state. Moreover, the only one patient with vertebral fractures showed one of the lower levels of TBS and the highest of BSI of the whole group, underling the importance of bone quality assessment in chronic illnesses.

The relation found between BMAD, BMAD Z-score, TBS, BSI and Tanner stages underlines the early onset of this phenomenon and the possible role of hormone replacement therapy for pubertal induction in these patients.

This study has some intrinsic limits due to the small number of patients, as expected for a single-centre study on a rare disease with a reported incidence of 11 per 1.000.000 individuals [39].

This study, being the first reporting data on pubertal delay in different forms of EB, emphasises the role of the Endocrinologist in the management and follow-up of growth impairment and pubertal development in this disease. Even in patients showing the first signs of pubertal development at an appropriate age, Tanner stages should be correctly investigated at every clinical assessment (every 6 months) in order to detect any eventual derangement of pubertal progression. Indeed, pubertal arrest, defined as failure to complete puberty within five years from its start, is frequently encountered in chronic illnesses. A prospective study on pubertal induction in those patients with severe pubertal delay is currently ongoing to find whether the association of hormone replacement to conventional treatment and nutritional support can improve growth and pubertal outcomes optimizing adult height, peak bone mass achievement and quality of life.

## Supporting information

S1 File Sexual maturation self-assessment tool [40]. (DOCX)
