# Supplementary figures and images for: A single-centre study on predictors and determinants of pubertal delay and growth impairment in Epidermolysis Bullosa
Source: PLoS One. 2022 Sep 6;17(9):e0274072. doi: 10.1371/journal.pone.0274072 (PMC9447886; doi:10.1371/journal.pone.0274072)

Supp.1 [40]
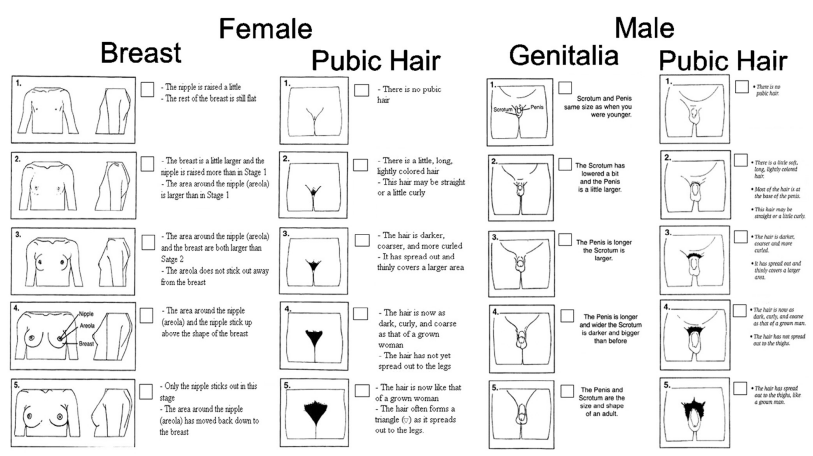

Supplement: S1 File — (DOCX) [file pone.0274072.s001.docx]
